# Use of evidence based practices to improve survival without severe morbidity for very preterm infants: results from the EPICE population based cohort

**DOI:** 10.1136/bmj.i2976

**Published:** 2016-07-05

**Authors:** Jennifer Zeitlin, Bradley N Manktelow, Aurelie Piedvache, Marina Cuttini, Elaine Boyle, Arno van Heijst, Janusz Gadzinowski, Patrick Van Reempts, Lene Huusom, Tom Weber, Stephan Schmidt, Henrique Barros, Dominico Dillalo, Liis Toome, Mikael Norman, Beatrice Blondel, Mercedes Bonet, Elisabeth S Draper, Rolf F Maier, and the EPICE Research Group

**Affiliations:** 1 INSERM UMR 1153, Obstetrical, Perinatal and Pediatric Epidemiology Research Team (Epopé), Center for Epidemiology and Statistics Sorbonne Paris Cité, DHU Risks in pregnancy, Paris Descartes University, Paris, 75014, France; 2 Department of Health Sciences, University of Leicester, Leicester, UK; 3 Clinical Care and Management Innovation Research Area, Bambino Gesù Children’s Hospital, Rome, Italy; 4 Department of Neonatology, Radboud University Medical Centre, Nijmegen, Netherlands; 5 Department of Neonatology, Poznan University of Medical Sciences, Poznan, Poland; 6 Department of Neonatology, Antwerp University Hospital, Antwerp; and Study Centre for Perinatal Epidemiology, Flanders, Brussels, Belgium; 7 Department of Obstetrics, Hvidovre University Hospital, Hvidovre, Denmark; 8 Department of Obstetrics, University Hospital, Philipps University, Marburg, Germany; 9 EPIUnit-Institute of Public Health, University of Porto, Porto, Portugal; 10 Health Department, Regione Lazio, Rome, Italy; 11 Unit of Neonates and Infants, Tallinn Children’s Hospital, Tallinn, Estonia; and University of Tartu, Tartu, Estonia; 12 Department of Clinical Science, Intervention and Technology, Division of Paediatrics, Karolinska Institute, Stockholm, Sweden; and Department of Neonatal Medicine, Karolinska University Hospital, Stockholm, Sweden; 13 Children’s Hospital, University Hospital, Philipps University, Marburg Germany

## Abstract

**Objectives** To evaluate the implementation of four high evidence practices for the care of very preterm infants to assess their use and impact in routine clinical practice and whether they constitute a driver for reducing mortality and neonatal morbidity.

**Design** Prospective multinational population based observational study.

**Setting** 19 regions from 11 European countries covering 850 000 annual births participating in the EPICE (Effective Perinatal Intensive Care in Europe for very preterm births) project.

**Participants** 7336 infants born between 24+0 and 31+6 weeks’ gestation in 2011/12 without serious congenital anomalies and surviving to neonatal admission.

**Main outcome measures** Combined use of four evidence based practices for infants born before 28 weeks’ gestation using an “all or none” approach: delivery in a maternity unit with appropriate level of neonatal care; administration of antenatal corticosteroids; prevention of hypothermia (temperature on admission to neonatal unit ≥36°C); surfactant used within two hours of birth or early nasal continuous positive airway pressure. Infant outcomes were in-hospital mortality, severe neonatal morbidity at discharge, and a composite measure of death or severe morbidity, or both. We modelled associations using risk ratios, with propensity score weighting to account for potential confounding bias. Analyses were adjusted for clustering within delivery hospital.

**Results** Only 58.3% (n=4275) of infants received all evidence based practices for which they were eligible. Infants with low gestational age, growth restriction, low Apgar scores, and who were born on the day of maternal admission to hospital were less likely to receive evidence based care. After adjustment, evidence based care was associated with lower in-hospital mortality (risk ratio 0.72, 95% confidence interval 0.60 to 0.87) and in-hospital mortality or severe morbidity, or both (0.82, 0.73 to 0.92), corresponding to an estimated 18% decrease in all deaths without an increase in severe morbidity if these interventions had been provided to all infants.

**Conclusions** More comprehensive use of evidence based practices in perinatal medicine could result in considerable gains for very preterm infants, in terms of increased survival without severe morbidity.

## Introduction

Very preterm infants—those born before 32 weeks’ gestation—represent fewer than 2% of all births but up to half of infant deaths.^1^ For survivors, the risks of cerebral palsy, visual and auditory deficits, cognitive impairments, psychiatric disorders, and behavioural problems are much higher than for children born at term.^2^ Ensuring the best outcomes for very preterm infants is essential for their future health and development and for reducing the burden for families and healthcare and social systems.

Wide disparities in the risk adjusted mortality and morbidity of very preterm infants across countries and neonatal units suggests that substantial gains are possible using current medical knowledge.^3^ ^4^ ^5^ ^6^ ^7^ Research comparing the care of very preterm infants across countries and units supports this assertion, as practices are not always consistent with the latest scientific evidence, including non-use of treatments shown to be effective and safe and use of others for which evidence is limited or where safety is of concern.^8^ ^9^ ^10^ ^11^ ^12^ ^13^

The promotion of applied evidence based care may thus be an important driver for achieving better outcomes in this high risk population, as shown in other areas of medicine.^14^ ^15^ ^16^ ^17^ Research from many medical specialties has highlighted the challenges of translating even convincing scientific knowledge into practice because of organisational, cultural, or personal barriers.^18^ ^19^ ^20^ Moreover, although evidence based interventions are shown to be effective in clinical trials, the selection criteria applied to achieve equipoise and ensure rigorous implementation of the protocol may limit the generalisability of results to the overall population of patients. It is thus necessary to improve knowledge on the use of interventions by clinicians and health planners and their impact in unselected populations.

The EPICE (Effective Perinatal Intensive Care in Europe) project established a population based cohort of very preterm infants in 19 regions in 11 European countries to investigate the use of evidence based practices and their association with outcomes in real life clinical settings. We investigated the use of four practices that have a high level of evidence for the care of very preterm infants and measured their association with mortality or neonatal morbidity, or both.

### Methods

#### Study design

The EPICE cohort is a geographically defined prospective study of all very preterm stillborn and liveborn infants from 22+0 weeks to 31+6 weeks of gestation, delivered in all public and private maternity hospitals in 19 regions in 11 European countries covering over 850 000 births annually: Belgium (Flanders), Denmark (eastern region), Estonia (entire country), France (Burgundy, Ile-de-France, and the northern region), Germany (Hesse and Saarland), Italy (Emilia-Romagna, Lazio, and Marche), the Netherlands (central and eastern region), Poland (Wielkopolska), Portugal (Lisbon and northern region), Sweden (greater Stockholm), and the United Kingdom (East Midlands, northern, and Yorkshire and Humber regions). Regions were selected for geographical and organisational diversity, feasibility (on-site infrastructure and expertise for implementing the protocol), and sample size considerations. Data were collected on births occurring between April 2011 and September 2012; in each region inclusions occurred over 12 months, except in France (six months).

Investigators abstracted data from medical records in obstetrical and neonatal units using a pretested standardised questionnaire with common definitions. Gestational age was defined as the best obstetric assessment based on information on last menstrual period and antenatal ultrasound examinations, which are part of routine obstetrical care in all regions. When there were several estimates, we used the following hierarchy to determine gestational age for the study: in vitro fertilisation treatment, ultrasound result based on earliest estimate, last menstrual period, fundal height measurement, and neonatal assessment at birth. We cross checked inclusions against delivery ward registers or another external data source. Infants were followed up until discharge home from hospital or into long term care or death.

### Patient involvement

The EPICE study included a European parent organisation in stakeholder meetings about the project’s preliminary results and analyses, including this study. EPICE maintains contact with parents in the cohort through regional newsletters, letters, and its website. A European parent organisation is part of our consortium for follow-up studies of the cohort.

### Study population

The study population comprised all infants without severe congenital anomalies born at 24+0 to 31+6 weeks’ gestation and admitted to a neonatal unit (n=7336 infants delivered in 335 maternity units and admitted to 242 neonatal units). We excluded infants born before 24 weeks’ gestation because there is no consensus across the regions about active treatment for these births^21^ (n=301 live births). Infants with severe congenital anomalies were excluded because of regional differences in screening and termination policies (n=126).^22^ We also excluded infants who died on the labour ward (n=112 ≥24 weeks’ gestation) because the EPICE database does not contain information on the degree of emergency in these cases, condition at birth, or neonatal resuscitation practices, and we were concerned that these cases were often situations where there was no opportunity to provide evidence based care. Furthermore, we surmised that reverse causality could be present—that is, a decision against active management could explain non-implementation of evidence based practices. Finally, we excluded out of hospital births that were unlikely to result in evidence based care (n=26).

### Definition of evidence based care: using an all or none approach

We used an all or none approach to study the use of evidence based practices. In contrast with an item by item assessment of performance or the creation of a composite measure, this approach considers whether all measures have been provided to each eligible patient.^23^ A restrained set of indicators is selected that should measure performance on the specified elements of good care and be related to the desired outcomes.^23^ The EPICE protocol included 17 practices with varying levels of evidence, from which we identified four with a high level of evidence that are related to neonatal mortality and morbidity and that could be measured reliably using information from medical records (see supplementary table S1). Some evidence based practices included in EPICE were not retained because they are evaluated with respect to longer term outcomes, such as screening for retinopathy of prematurity. Others were not selected because the evidence is not of highest quality and therefore unlikely to be consensually adopted in all regions, such as active management of patent ductus arteriosus.^24^ We then established minimum thresholds for evidence based care that would be accepted across all regions.

Selected indicators were: delivery in a maternity unit with appropriate neonatal care services^25^ using national level of care designations (see supplementary table S2); any administration of antenatal corticosteroids before delivery^26^; effective prevention of hypothermia, defined as a temperature on admission of 36°C or more that corresponds to the lower limit of current recommendations^27^ ^28^; and surfactant used within two hours after birth or early nasal continuous positive airway pressure for infants born before 28 weeks’ gestation.^29^ ^30^ We computed a variable measuring the receipt of all practices, given each infant’s eligibility.

### Outcomes

Our outcomes were in-hospital mortality, defined as death before discharge home or into long term paediatric care; severe neonatal morbidity among infants discharged alive; and a composite of in-hospital mortality or severe neonatal morbidity, or both. Severe neonatal morbidity comprised intraventricular haemorrhage grade III or IV, cystic periventricular leukomalacia, retinopathy of prematurity stages III to V, and severe necrotising enterocolitis. Intraventricular haemorrhage grades were determined using Papile’s classification,^31^ and we recorded periventricular leukomalacia only if cystic abnormalities were present on ultrasound or magnetic resonance imaging scans. Severe necrotising enterocolitis was assessed by surgery or peritoneal drainage because Bell stages were not routinely recorded in all regions. We did not include bronchopulmonary dysplasia because large regional variability in respiratory management and oxygen saturation targets affect rates of this outcome variable.^32^

### Covariables

We identified clinical and healthcare factors likely to influence both the probability of receiving evidence based care and our outcomes based on the scientific literature and biological plausibility. These factors included gestational age, sex, multiple pregnancy, pregnancy complications (preterm premature rupture of membranes, eclampsia or pre-eclampsia, and haemolysis, elevated liver enzymes, low platelets (HELLP) syndrome), small for gestational age , type of delivery (prelabour caesarean section, intrapartum caesarean section, vaginal), and Apgar score at five minutes. We categorised small for gestational age as less than the third and less than the 10th centile for gestational age and sex with Hadlock’s references adapted to national population values using Gardosi’s model.^33^ We also identified cases where the birth was more likely to have been unexpected, with a rapid onset precluding use of evidence based practices, defined by delivery on the same day as maternal admission to hospital without in utero transfer. We also assessed neonatal transport in the first 48 hours after delivery; this variable was not included in multivariable models, but used for sensitivity analyses to identify infants who were born in the maternity unit associated with the neonatal unit where they received care (termed inborn). For the propensity score analysis we identified other possible confounders (see below).

### Missing data

Most variables had low proportions of missing data: less than 1% (gestational age, birth weight, sex, multiple pregnancy, neonatal transport, antenatal corticosteroids), 1-3% (pregnancy complications, mode of delivery, neonatal morbidity), and 4-5% (Apgar score and admission to delivery time). In contrast, admission temperature was missing in 12.1% of cases (n=886). We used multiple imputations chained equations to impute missing data, based on all variables in the study.^34^ We used 100 imputed datasets.^35^ Outcomes were not imputed. Results are presented using the imputed data; however, models using list-wise deletion are included as supplementary tables.

### Analysis strategy

We described the use of each evidence based practice and investigated the factors associated with our all or none composite (full evidence based care). We then investigated the association of full evidence based care with our three primary outcome variables. For both analyses, we used generalised linear models to take into consideration the clustering of births within hospitals, assuming a Poisson distribution with robust standard errors to estimate risk ratios.^36^ Region was included as a fixed effect.

To assess the impact of use of evidence based care on outcomes, we developed propensity scores to control for observed confounding factors. Probabilities of receiving full evidence based care were generated using logistic regression models, including investigator selected covariables and other possible confounders (24 variables in total and four interactions, see supplementary table S3). Missing values for these additional variables were included as a separate category. After consideration of the propensity score using standardised differences, we assessed the balance in our covariables between infants receiving and not receiving evidence based care. The propensity score was included in our primary analyses by weighting each infant by the inverse propensity of his or her group.^37^ We compared results from propensity score models with those from a multivariable model, adjusting for investigator selected covariables. We ran our models for all infants and inborn infants born the day after the mother had been admitted to hospital.

To check that our findings did not reflect the influence of only one of the evidence based practices, we reran our models four times, removing each indicator in turn. We also assessed the impact of non-receipt of one versus two or three, or four practices, compared with receiving all interventions, using the multivariable model with investigator selected covariables. To estimate the impact of receipt of evidence based care, we predicted cases of death and severe morbidity if all infants and almost all infants received the evidence based practices for which they were eligible by keeping coefficients and variables values constant and setting the evidence based care variable to “yes” for all infants, or “yes” for 90% of the infants. The 10% of infants assigned to continued non-evidence based care in the latter model were those with the lowest propensity scores, reflecting their likelihood of receiving evidence based care based on their characteristics.

Analyses were carried out using STATA 13.0 SE (Stata, College Station, TX).

## Results

The mean gestational age in our sample was 28.7 weeks, with a mean birth weight of 1224 g (table 1); 25.0% (n=1835) of births were preceded by preterm premature rupture of membranes and 42.7% (n=3130) were prelabour caesarean sections; 21.9% (n=1605) of infants were born on the same day as maternal admission to hospital without in utero transfer; 11.0% (n=808) were transported to another hospital after birth. In-hospital mortality was 9.2% (n=672), and 10.3% (n=669/6479) of survivors had a severe neonatal morbidity.

**Table 1 tbl1:** Clinical characteristics and care of very preterm infants admitted for neonatal care. Values are numbers (percentages) unless stated otherwise

Indicators	Neonatal admissions (n=7336)
Mean (SD) gestational age (weeks)	28.7 (2.1)
Mean (SD) birth weight (g)	1224.0 (383.9)
Gestational age (weeks):	
24-26	1372 (18.7)
27-29	2652 (36.2)
30-31	3312 (45.1)
Male	3957 (53.9)
Multiples	2300 (31.4)
Small for gestational age*	2380 (32.4)
Pre-eclampsia, eclampsia, or HELLP	1153 (15.7)
Preterm premature rupture of membranes	1835 (25.0)
Prelabour caesarean section	3130 (42.7)
Intrapartum caesarean section	1868 (25.5)
Vaginal delivery	2339 (31.9)
Apgar score <7 at 5 minutes	1244 (17.0)
Organisation of delivery:	
In utero transfer (IUT)	2147 (29.3)
Delivery on day of maternal admission, no IUT	1605 (21.9)
Neonatal transport in first 48 hours	808 (11.0)
Mortality and morbidity:	
In-hospital mortality	672 (9.2)
Any severe morbidity (survivors to discharge)	669 (10.3)
Intraventricular haemorrhage grade III or IV or cystic periventricular leukomalacia	407 (6.2)
Retinopathy of prematurity grades III-V	234 (3.6)
Necrotising enterocolitis with surgery	116 (1.8)
Death or severe morbidity	1341 (18.8)
Infants included by region:	
Belgium: Flanders	712 (9.7)
Denmark: eastern	324 (4.4)
Estonia	150 (2.0)
France:	
Northern	293 (4.0)
Burgundy	89 (1.2)
Ile-de-France	816 (11.1)
Germany:	
Hesse	555 (7.6)
Saarland	132 (1.8)
Italy:	
Lazio	536 (7.3)
Emilia	419 (5.7)
Marche	101 (1.4)
Netherlands: east-central	368 (5.0)
Poland: Wielkopolska	259 (3.5)
Portugal:	
Northern	274 (3.7)
Lisbon	424 (5.8)
United Kingdom:	
Northern	406 (5.5)
East Midlands	545 (7.4)
Yorkshire and Humber	691 (9.4)
Sweden: Stockholm	242 (3.3)

HELLP=haemolysis, elevated liver enzymes, and low platelets.

*Birth weight less than 10th centile of intrauterine references.

Most infants received at least one of the evidence based practices (fig 1): 88.2% (n=6468) for appropriate place of birth, 89.2% (n=6541) for antenatal steroids, 74.4% (5455) for an admission temperature of 36°C or more, and 83.0% (n=6086) for surfactant used within two hours or early nasal continuous positive airway pressure. However, only 58.3% (n=4275) of infants received all four practices and 9.6% (n=704) did not receive at least two of the practices. The probability of receiving full evidence based care was lower for infants of less than 26 weeks gestational age, singletons, small for gestational age infants, infants with low Apgar scores (<7 at five minutes), infants transported after birth, and infants born on the day of maternal admission (table 2). Full evidence based care by region ranged from 32.0% to 75.5%, and differences remained statistically significant after adjustment for clinical and delivery characteristics.

**Figure f1:**
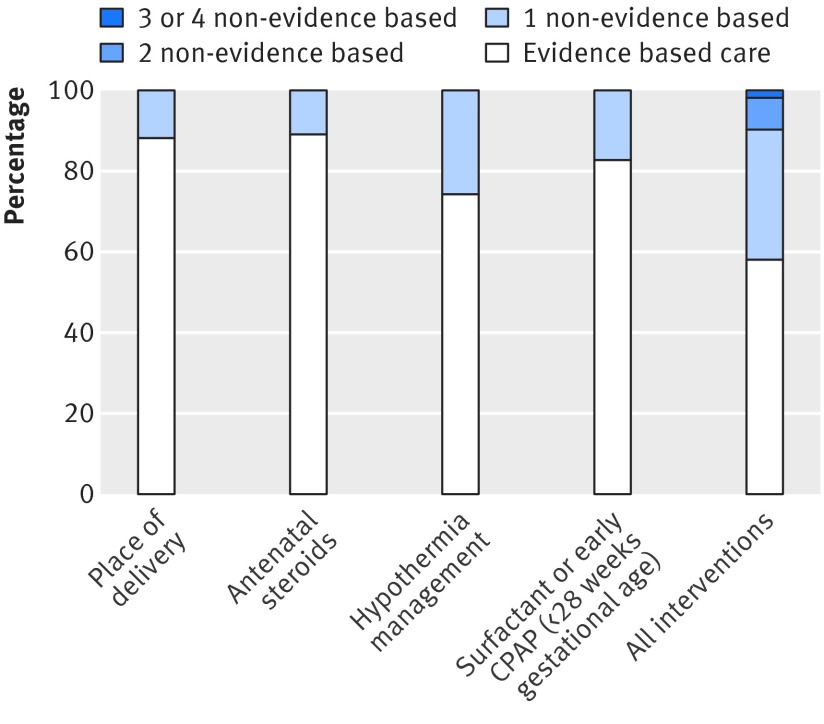
**Fig 1** Receipt of each evidence based intervention in all or none composite as well as receipt of all interventions among infants of 24+0 to 31+6 weeks’ gestation admitted to neonatal care. CPAP=continuous positive airway pressure

**Table 2 tbl2:** Use of four evidence based practices by clinical and care characteristics

Characteristics	All evidence based practices (%)	Crude risk ratio (95% CI)	Adjusted risk ratio* (95% CI)
Gestational age (weeks):			
24-26	39.2	0.62 (0.55 to 0.70)	0.62 (0.55 to 0.69)
27-29	61.3	0.97 (0.93 to 1.02)	0.96 (0.92 to 1.01)
30-31	63.8	Reference	Reference
Sex:			
Male	58.7	1.01 (0.98 to 1.05)	1.01 (0.97 to 1.05)
Female	57.8	Reference	Reference
Type of pregnancy:			
Singleton	55.7	Reference	Reference
Multiple	63.9	1.17 (1.11 to 1.22)	1.13 (1.08 to 1.18)
Small for gestational age (by centile):			
<3rd	53.5	0.90 (0.84 to 0.97)	0.88 (0.82 to 0.94)
3-<10th	57.5	0.96 (0.90 to 1.02)	0.92 (0.87 to 0.97)
≥10th	59.9	Reference	Reference
Preterm premature rupture of membranes	67.7	1.23 (1.17 to 1.29)	1.13 (1.08 to 1.19)
Pre-eclampsia, eclampsia, or HELLP	53.7	0.91(0.84 to 0.99)	0.94 (0.87 to 1.01)
Type of delivery:			
Prelabour caesarean section	57.8	1.05 (0.98 to 1.12)	1.04 (0.98 to 1.10)
Intrapartum caesarean section	61.6	1.09 (1.02 to 1.16)	1.03 (0.97 to 1.10)
Vaginal	56.3	Reference	Reference
Apgar score at 5 minutes:			
<7	48.9	0.79 (0.74 to 0.85)	0.89 (0.84 to 0.95)
≥7	60.2	Reference	Reference
Delivery:			
>1 day of maternal admission	63.2	1.60 (1.47 to 1.75)	1.59 (1.46 to 1.73)
Same day as maternal admission	42.2	Reference	Reference
Regions†			
Belgium: Flanders	59.0	1.04 (0.87 to 1.24)	0.99 (0.85 to 1.15)
Denmark: eastern	49.6	0.87 (0.55 to 1.38)	0.88 (0.55 to 1.41)
Estonia	75.4	1.32 (1.06 to 1.64)	1.36 (1.14 to 1.62)
France:			
Northern	46.4	0.81 (0.63 to 1.06)	0.82 (0.63 to 1.05)
Burgundy	68.5	1.20 (0.85 to 1.69)	1.14 0.88 to 1.48)
Ile-de-France	48.4	0.85 (0.73 to 0.99)	0.84 (0.73 to 0.96)
Germany:			
Hesse	73.4	1.29 (1.16 to 1.43)	1.27(1.15 to 1.39)
Saarland	62.7	1.10 (0.87 to 1.39)	1.09 (0.86 to 1.38)
Italy:			
Lazio	42.4	0.75 (0.57 to 0.98)	0.72 (0.55 to 0.95)
Emilia	67.9	1.19 (1.08 to 1.32)	1.18 (1.08 to 1.29)
Marche	51.5	0.90 (0.76 to 1.07)	0.93 (0.78 to 1.11)
Netherlands: east-central	49.2	0.86 (0.70 to 1.07)	0.84 (0.69 to 1.03)
Poland: Wielkopolska	53.9	0.95 (0.54 to 1.66)	0.94 (0.58 to 1.53)
Portugal:			
Northern	47.1	0.83 (0.63 to 1.10)	0.81 (0.61 to 1.09)
Lisbon	32.1	0.56 (0.42 to 0.75)	0.55 (0.42 to 0.73)
United Kingdom:			
Northern	62.6	1.10 (0.83 to 1.46)	1.16 (0.88 to 1.53)
East Midlands	75.5	1.33 (1.19 to 1.47)	1.40 (1.27 to 1.54)
Yorkshire and Humber	75.0	1.32 (1.17 to 1.48)	1.39(1.25 to 1.55)
Sweden: Stockholm	68.8	1.21 (1.02 to 1.43)	1.24 (0.99 to 1.55)

HELLP=haemolysis, elevated liver enzymes, and low platelets.

*Adjusted for gestational age, sex, small for gestational age, multiple pregnancy, pregnancy complications, type of delivery, Apgar score, born on same day as maternal admission without in utero transfer.

†Sample average was used as reference for deriving risk ratio for each region.

In unadjusted comparisons, mortality and severe morbidity were lower for infants with full evidence based care (table 3). We generated propensity scores ranging from 2% to 97%, which achieved balance in our covariables (see supplementary table S3). The area under the receiver operating characteristic curve for the propensity score model was 0.76 (95% confidence interval 0.75 to 0.77). In propensity score weighted models, mortality for infants receiving all evidence based practices was 28% lower (risk ratio 0.72, 95% confidence interval 0.60 to 0.87) and mortality or severe morbidity was 18% lower (0.82, 0.73 to 0.92). Results were similar in models adjusting for investigator selected covariables and when the analysis was restricted to inborn infants delivered the day after the mother had been admitted to hospital. Sensitivity analyses with different combinations of the evidence based practices confirmed that one indicator was not driving these associations, and using list-wise deletion of missing data yielded similar results (see supplementary table S4 and fig S1).

**Table 3 tbl3:** In-hospital mortality and severe morbidity by receipt of all evidence based practices. Values are numbers/total numbers (percentages) unless stated otherwise

Variables	In-hospital mortality (all neonatal admissions)	Severe morbidity (survivors to discharge)	Mortality or severe morbidity (all neonatal admissions)
All infants	672/7336 (9.2)	669/6479 (10.3)	1341/7151 (18.8)
Not receiving evidence based care	431/3060 (14.1)	350/2552 (13.7)	780/2982 (26.2)
Receiving evidence based care	241/4276 (5.6)	319/3927 (8.1)	561/4169 (13.5)
Crude risk ratio (95% Cl)	0.42 (0.35 to 0.50)	0.56 (0.48 to 0.65)	0.51 (0.45 to 0.57)
Adjusted risk ratio* (95% Cl)	0.72 (0.60 to 0.87)	0.82 (0.71 to 0.94)	0.81 (0.72 to 0.90)
Propensity weighted risk ratio† (95% CI)	0.72 (0.60 to 0.87)	0.87 (0.75 to 1.02)	0.82 (0.73 to 0.92)
Inborn infants, excluding deliveries on same day as admission‡	464/5293 (8.8)	458/4695 (9.8)	911/5158 (17.9)
Not receiving evidence based care	282/1905 (14.8)	214/1579 (13.6)	495/1860 (26.6)
Receiving evidence based care	182/3388 (5.4)	244/3116 (7.8)	427/3298 (12.9)
Crude risk ratio (95% Cl)	0.39 (0.31 to 0.49)	0.54 (0.45 to 0.66)	0.49 (0.42 to 0.56)
Adjusted risk ratio (95% Cl)	0.72 (0.57 to 0.92)	0.83 (0.69 to 1.00)	0.81 (0.71 to 0.93)
Propensity weighted risk ratio† (95% CI)	0.69 (0.55 to 0.87)	0.87 (0.72 to 1.06)	0.80 (0.70 to 0.92)

*Adjusted for gestational age, sex, small for gestational age, multiple pregnancy, pregnancy complications, type of delivery, Apgar score, born on same day as maternal admission without in utero transfer, and region.

†Adjusted for gestational age.

‡Infants admitted to hospital in neonatal unit in same hospital as maternity unit (no neonatal transport in first 48 hours).

We also analysed whether there was a dose-response effect, whereby infants receiving fewer evidence based practices had worse outcomes. In adjusted models, compared with infants receiving all evidence based practices, receiving one fewer practice was associated with a risk ratio of mortality of 1.32 (1.09 to 1.60), two fewer of 1.55 (1.23 to 1.95), and three or four fewer of 1.81 (1.26 to 2.61). These estimates for death or severe neonatal morbidity, or both were 1.20 (1.10 to 1.34), 1.32 (1.12 to 1.44, and 1.59 (1.30 to 1.94), respectively. Most infants not receiving evidence based care, however, received only one fewer practice (77.0%).

Table 4 illustrates the potential impact of providing evidence based care more broadly. We simulated two different situations for eligible infants: one where evidence based care was provided to all infants, and one where such care was provided to 90%. The model with evidence based care provided to all infants predicted a reduction of 28.1% of the 432 deaths in the group that did not receive evidence based care, which represents a reduction of 17.9% of all deaths. Of the 781 cases of death or severe morbidity, or both in the non-evidence based group, the reduction was estimated at 19.4%, corresponding to 11.3% of the total 1341 cases. In the scenario where 90% of infants received full evidence based care, 18.3% of deaths in the non-evidence based group would be prevented, representing 11.8% of all deaths. The percentages for mortality or severe morbidity were 12.1% and 7.0%, respectively.

**Table 4 tbl4:** Predicted deaths and cases of severe morbidity if all infants or 90% of infants received all evidence based practices

Variables	No of events (%)			
	Receiving evidence based care	Not receiving evidence based care	Total	Reduction*
Observed:				
Deaths	241 (5.6)	431 (14.1)	672 (9.2)	
Severe morbidity†	319 (8.1)	350 (13.7)	669 (10.3)	
Death and/or severe morbidity	561 (13.4)	780 (26.2)	1341 (18.8)	
If all infants received evidence based care‡:				
Deaths	241 (5.6)	310 (10.1)	552 (7.5)	120 (17.9)
Severe morbidity†	319 (8.1)	285 (11.1)	604 (9.3)	65 (9.7)
Death and/or severe morbidity	561 (13.4)	629 (21.1)	1190 (16.6)	151 (11.3)
If 90% infants received evidence based care‡§:				
Deaths	241 (5.6)	352 (11.5)	593 (8.1)	79 (11.8)
Severe morbidity†	319 (8.1)	310 (12.1)	629 (9.7)	40 (6.0)
Death and/or severe morbidity	561 (13.4)	686 (23)	1247 (17.4)	94 (7.0)

*Number and percentage of events avoided (total observed−total predicted.

†Survivors only.

‡Number of deaths and severe morbid events predicted from final model, adjusting for investigator selected covariables.

§10% of sample who were assumed to not have evidence based care were those with lowest propensity scores—that is, least likely to receive evidence based care because of their clinical or healthcare characteristics.

## Discussion

Only 58.3% of very preterm infants received all of four high evidence practices for which they were eligible, including delivery in a maternity unit with appropriate level of neonatal care, administration of antenatal corticosteroids, prevention of hypothermia, and surfactant used within two hours of birth or early nasal continuous positive airway pressure. In-hospital mortality as well as a combined outcome of mortality or severe neonatal morbidity, or both were lower for infants who received all components of evidence based care in our all or none measure. These results suggest that more comprehensive use of these high evidence and widely accessible practices could yield substantial gains in survival without severe morbidity for these infants at high risk.

### Strengths and limitations of this study

The strengths of our study are its large and heterogeneous multiregional population based sample, including public and private healthcare facilities, which ensure the generalisability of our results to a wide range of settings. The EPICE study also developed common study instruments and protocols to obtain comparable high quality data across regions. Our study also has limitations. It was challenging to define evidence based practices that were adapted to diverse cultural and organisational settings and could be identified from data systematically available in medical records; we thus selected the most liberal criteria to ensure high acceptability of our thresholds in all contexts. Others may prefer more stringent thresholds—for example, higher temperatures on admission, or administration of full courses of antenatal steroids. Although choosing conservative cut-off points overestimates the use of evidence based care, it does not invalidate our main finding of low use of these practices and the gains associated with the improvement in evidence based perinatal management. We excluded deaths on the labour ward because these were more likely to result from emergency situations where there was no opportunity to arrange a maternal transfer or to administer antenatal steroids and also because of concerns with reverse causality. However, suboptimal use of evidence based care probably contributes to the risks of death on the labour ward, leading us to underestimate total effects; future studies with data on exact timing of maternal arrival at the hospital, resuscitation practices in the delivery room, and parental opinions are needed to explore this issue further. We had some missing data on interventions and, in particular, on admission temperature. These cases may reflect less focus on hypothermia prevention and have led to an overestimation of evidence based care and an underestimation of the impact on outcomes. Finally, our study only included short term neonatal outcomes; the longer term impacts on child neurodevelopment and other measures of child and family wellbeing are an important area for further investigation.

### Comparison with other studies

An all or none approach makes it possible to evaluate the process of care and the potential for improvement for high evidence based interventions that are already widely used. We identified four interventions for the care of very preterm infants, which are supported by evidence, linked to better health outcomes, and could be measured in our study in a standardised way. Two of these refer to the management of pregnant women with threatened preterm delivery and two to the early management of infants. This selection reflects our conviction that optimal outcomes for very preterm infants require both prenatal and postnatal interventions.

Our all or none composite comprises practices that have been shown in meta-analyses of randomised controlled trials or observational studies to improve outcome and have been accepted as standard care for over a decade. For the first practice—delivery in an appropriate maternity unit—meta-analyses of observational studies have shown that birth in a maternity unit with on-site neonatal intensive care (often termed a level 3 unit) is associated with better outcomes for very preterm infants.^25^ As the specialisation of units by level of care differs in Europe,^38^ we used regional guidelines to identify appropriate units. For the second practice—administration of antenatal corticosteroids—meta-analyses have shown reductions in neonatal death, respiratory distress syndrome, intraventricular haemorrhage, necrotising enterocolitis, and systemic infections.^26^ Our third practice focused on hypothermia at admission to the neonatal unit, which is associated with higher mortality and morbidity.^28^ ^39^ Plastic wraps or bags, plastic caps, skin-to skin-contact, and transwarmer mattresses have all been shown to be effective for the prevention of hypothermia.^40^ As the combination of these measures can vary between units, we considered a non-hypothermic temperature at neonatal admission to indicate use of effective evidence based practices. Multiple definitions of hypothermia are used (<36.0°C or 36.5C°)^27^ ^28^; to ensure consensus on our thresholds, we used the more liberal definition of 36.0°C. Our last practice focused on respiratory management for extremely preterm infants, and it is based on two recent meta-analyses. One showed less chronic lung disease or death when using early stabilisation on nasal continuous positive airway pressure, with selective surfactant administered to infants requiring intubation.^30^ The other showed less acute and chronic pulmonary injury and neonatal mortality from surfactant administered within the first two hours of life in infants intubated for respiratory distress.^29^ Given these results, we judged either early administration of surfactant or early nasal continuous positive airway pressure in infants born less than 28 weeks of gestation to be evidence based interventions. This combined criterion is in accordance with European consensus statements.^41^ ^42^

We found high rates for use of each practice—between 75% and 90%, corroborating network and single country studies.^43^ ^44^ The population receiving full evidence based care was, however, much lower: fewer than 60% of infants, revealing more severe deficits in the care process. We further illustrated the high impact on population health of implementing all these practices by simulating situations in which all and almost all infants received the evidence based practices for which they were eligible. While we observed a dose-response association related to the number of practices not administered, most infants received only one fewer than the total. These findings underscore the limits of evaluating practices in isolation and support the growing focus in other clinical areas of medicine and other specialties, including adult care, on bundling effective practices to improve processes of care and to achieve best outcomes.^45^ ^46^

While it seems surprising that such a low proportion of infants received these key elements of care, our results corroborate research from many disciplines showing the difficulty of translating effective interventions into routine clinical practice. Barriers include the doctors’ education, knowledge, and attitudes,^20^ and organisational obstacles within the unit, such as lack of strong leadership, absence of written protocols, absence of in-service training, no management support, and the size of the facility.^47^ ^48^ Differences in ethical attitudes influencing active management of extremely preterm infants may be another contributing factor,^21^ although the exclusion of births under 24 weeks and labour ward deaths probably minimised this effect. Many countries recommend active management starting at 24 weeks of gestation, but in others this remains a grey area in which decisions on active management are discussed with parents.^49^ Finally, the regulatory context may be one driver of implementation for these interventions, although the relation between the existence of guidelines and practice is complex.^18^ ^20^ All these factors likely contribute to the variability in evidence based care observed between the European regions included in this study, corroborating previous reports of wide practice variability for the care of very preterm infants across countries and across hospitals within countries.^8^ ^10^ ^11^ ^12^ ^50^

Our results also showed that the organisational challenges of managing unexpected deliveries contributed to low use of evidence based care for very preterm infants, although this did not explain the shortfall in use of evidence based care that existed even for inborn babies whose mothers were admitted to hospital for at least one day before their birth. Further investigation is needed to assess whether actions targeting the organisation and provision of care in situations involving unexpected or precipitous deliveries could achieve rates of the all or none composite close to 100%.

### Conclusion and policy implications

Only 58.3% of very preterm infants admitted for perinatal care in 19 European regions received all of the four evidence based practices for which they were eligible; receipt of evidence based care was associated with improved survival after taking into consideration clinical and delivery factors that may affect access to care and outcomes. Maximising the number of very preterm infants who receive the complete set of these well proved practices could yield substantial gains in survival without increasing severe neonatal morbidity in survivors.

**What is already known on this topic**

Very preterm infants face high risks of mortality and severe neonatal morbidity compared with infants born at term
Effective perinatal interventions exist to improve survival and reduce neonatal morbidity
Country and unit variations in very preterm outcome are large and may reflect suboptimal use of evidence based care

**What this study adds**

Only 58.3% of very preterm infants admitted for neonatal care in 19 European regions received all of the four evidence based practices for which they were eligible
These very preterm infants had higher risk adjusted survival without severe morbidity, suggesting more comprehensive provision of evidence based practices could yield substantial gains
The study’s findings support the growing focus on bundling effective practices to improve processes of care and to achieve best outcomes

## Web extra

Web extra material supplied by author

**Appendix 1**: supplementary figure S1 and tables S1-S4

**Appendix 2**: List of hospitals participating in EPICE study

## Notes

We thank Elizabeth Howell, Michael Obladen, Raphael Porcher, and Philippe Ravaud for their input on draft versions of this manuscript (permission was requested for these acknowledgments), and the participation of the Departments of Obstetrics and Neonatology from hospitals in the EPICE regions (see supplementary file for list). EPICE Research Group: Belgium: Flanders (E Martens, G Martens, P Van Reempts); Denmark: eastern region (K Boerch, A Hasselager, L Huusom, O Pryds, T Weber); Estonia (L Toome, H Varendi); France: Burgundy, Ile-de France and northern region (P Y Ancel, B Blondel, A Burguet, P H Jarreau, P Truffert); Germany: Hesse (R F Maier, B Misselwitz, S Schmidt), Saarland (L Gortner); ITALY: Emilia Romagna (D Baronciani, G Gargano), Lazio (R Agostino, D DiLallo, F Franco), Marche (V Carnielli), M Cuttini; Netherlands: eastern and central (C Koopman-Esseboom, A van Heijst, J Nijman); Poland: Wielkopolska (J Gadzinowski, J Mazela); Portugal: Lisbon and Tagus Valley (L M Graça, M C Machado), northern region (Carina Rodrigues, T Rodrigues), H Barros; Sweden: Stockholm (A K Bonamy, M Norman, E Wilson); UK: East Midlands and Yorkshire and Humber (E Boyle, E S Draper, B N Manktelow), northern region (A C Fenton, D W A Milligan); INSERM, Paris (J Zeitlin, M Bonet, A Piedvache).

Contributors: JZ and RFM had full access to all of the data in the study and take responsibility for the integrity of the data and the accuracy of the data analysis. They act as guarantors of the study. JZ, BNM, MC, EB, AvH, JG, LH, TW, SS, PVR, HB, DD, LT, MN, BB, MB, ESD, and RFM conceived and designed the study. JZ, BM, AP, MC, EB, AvH, JG, LH, TW, SS, PVR, HB, DD, LT, MN, BB, MB, ESD, RFM, and all authors in EPICE Research Group acquired, analysed, or interpreted the data. JZ, BNM, AP, MC, EB, AvH, JG, LH, TW, SS, PVR, HB, DD, LT, MN, BB, MB, ESD, and RFM drafted the manuscript. All authors (including authors listed in EPICE Research Group) critically revised the manuscript for important intellectual content and gave approval of the final version of the manuscript. JZ, BNM, AP, and MB carried out the statistical analysis. JZ, BNM, and ESD supervised the study. GM, OP, LT, JZ, RFM, MC, DD, AvH, JG, HB, MN, and ESD obtained funding.

Ethical approval: Ethical approval was obtained in each region from regional or hospital ethics committees, as required by national legislation. The European study was also approved by the French Advisory Committee on Use of Health Data in Medical Research (CCTIRS) and the French National Commission for Data Protection and Liberties (CNIL).

Data sharing: The statistical code is available from the corresponding author (Jennifer.zeitlin@inserm.fr). Authorisations from our institutional review board do not allow us to share other data outside of the consortium.

Transparency: The lead author (JZ) affirms that this manuscript is an honest, accurate, and transparent account of the study being reported; that no important aspects of the study have been omitted.
