# Supplementary material for: Use of evidence based practices to improve survival without severe morbidity for very preterm infants: results from the EPICE population based cohort
Source: BMJ. 2016 Jul 5;354:i2976. doi: 10.1136/bmj.i2976 (PMC4933797; doi:10.1136/bmj.i2976)
Supplement: Supplementary file 1 — Appendix 1: supplementary figure S1 and tables S1-S4 [file zeij028369.ww1_default.pdf]

Supplementary Material

Figure S1 - Comparison of relative risks of adverse outcome associated with receipt of full EB perinatal care in models using imputed versus non-imputed data for missing observations

	log(RR) (95% CI)
**In-hospital mortality**	
Crude	
non-imputed	-0.38 [-0.46 ; -0.30]
imputed	-0.38 [-0.46 ; -0.30]
Adjusted	
non-imputed	-0.12 [-0.21 ; -0.03]
imputed	-0.14 [-0.22 ; -0.06]
Propensity weighted	
non-imputed	-0.11 [-0.21 ; -0.01]
imputed	-0.14 [-0.22 ; -0.06]
**Severe morbidity**	
Crude	
non-imputed	-0.25 [-0.32 ; -0.19]
imputed	-0.25 [-0.32 ; -0.19]
Adjusted	
non-imputed	-0.11 [-0.18 ; -0.05]
imputed	-0.09 [-0.15 ; -0.03]
Propensity weighted	
non-imputed	-0.09 [-0.16 ; -0.01]
imputed	-0.06 [-0.12 ; 0.01]
**Mortality or morbidity**	
Crude	
non-imputed	-0.29 [-0.35 ; -0.24]
imputed	-0.29 [-0.35 ; -0.24]
Adjusted	
non-imputed	-0.10 [-0.15 ; -0.04]
imputed	-0.09 [-0.14 ; -0.05]
Propensity weighted	
non-imputed	-0.09 [-0.15 ; -0.03]
imputed	-0.09 [-0.14 ; -0.04]

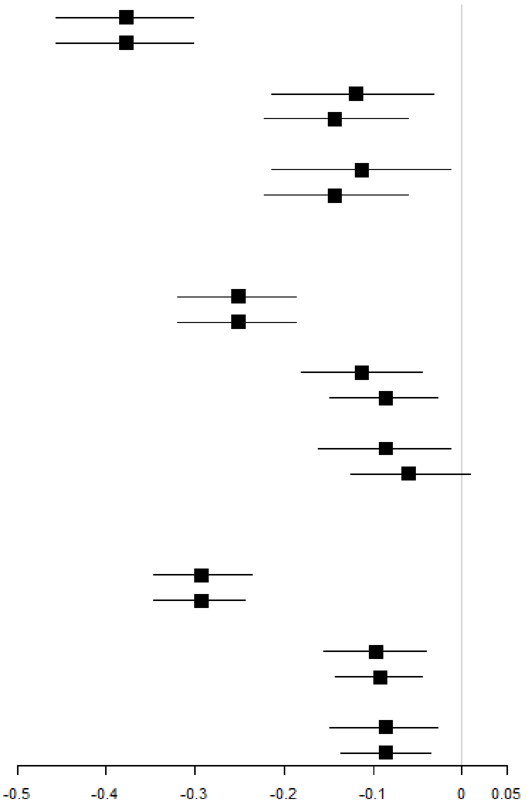

Table S1 - Practices/interventions included in the EPICE study and selection for the All-or-None composite

	Practice with high level of evidence	If yes: associated with mortality and short term morbidity in scientific literature	If yes: data collected on use at infant level
1. Delivery in maternity units with appropriate on-site neonatal intensive care services #	X	X	X
2. Antibiotics for preterm labour			
3. Use of tocolysis			
4. Administration of antenatal corticosteroids #	X	X	X
5. Magnesium sulphate as a neuroprotective			
6. Delivery by caesarean section for VPT			
7. Time (early or late) for cord clamping			
8. Hypothermia prevention #	X	X	X
9. Surfactant Replacement Therapy #	X	X	X
10. Inhaled Nitric Oxide (NO)			
11. Breastfeeding and breast milk use	X	X*	
12. Probiotic Use			
13. Management of patent ductus arteriosus (PDA)			
14. Kangaroo care (skin-to-skin)			
15. BPD prevention strategies (vitamin A/Caffeine)			
16. Postnatal corticosteroids (non use)	X		X
17. ROP screening and treatment	X		X

NOTE: * associated with necrotizing enterocolitis, data only available on use of BF for first enteral feed and at discharge. Not sufficient for measuring overall use during hospitalization. # Seleced for the All-or-None-Analysis

The EPICE study also collected information from units on parental visiting regulations as well as post-discharge follow-up programs, but these were not considered interventions.

Table S2 - Level of care designations by country used to identify appropriate maternity of birth

Belgium	Level III
Denmark	Level III and IIB for 28 weeks and over*
Estonia	Level III
France	Level III
Germany	Level III and level II for 29 weeks and over
Italy	Level III
Netherlands	Level III
Poland	Level III
Portugal	Level III
UK regions	Level III and level II for 27 weeks and over*
Sweden	Level III and level IIB for 28 weeks and over*

NOTE: Level II B units staffed and equipped to care for this population of very preterm infants

Table S3 – Variables included in propensity score and sample balance after weighting on score

Characteristics	Overall cohort			Inverse Probability-Weighted Cohort		
	Non allEB (N = 3061)	allEB (N = 4275)	Standardised difference	Non allEB (N = 7308)	allEB (N = 7302)	Standardised difference
Gestational age (wk), mean (SD)	28.2 (2.3)	29 (1.9)	35.5	28.7 (2.2)	28.7 (2.0)	1.7
Mother's age (y), mean (SD)	30.3 (6.1)	30.6 (6.0)	4.5	30.5 (6.1)	30.6 (6.0)	1.4
Length of stay (day), mean (SD)	4.7 (9.1)	6.1 (10.6)	15.1	5.4 (10.5)	5.6 (9.8)	1.9
	N (%)	N (%)		N (%)	N (%)	
Sex: Male	1636 (53.5)	2321 (54.3)	1.7	4037 (55.1)	3958 (54.1)	2.0
Type of pregnancy : Multiple	828 (27.1)	1473 (34.4)	16.0	2314 (31.6)	2323 (31.8)	0.4
SGA:						
<3rd	717 (23.4)	827 (19.3)	10.0	1526 (20.8)	1536 (21)	0.4
3-10th	357 (11.7)	480 (11.2)	1.4	852 (11.6)	865 (11.8)	0.6
≥10th	1986 (64.9)	2969 (69.4)	9.7	4944 (67.5)	4909 (67.2)	0.8
Previous caesarean section	335 (10.9)	509 (11.9)	3.0	842 (11.5)	824 (11.3)	0.7
Parity:						
Zero	1688 (55.2)	2405 (56.2)	2.2	4105 (56.1)	4088 (55.9)	0.3
One	755 (24.7)	1042 (24.4)	0.72	1794 (24.5)	1795 (24.6)	0.13
Two	342 (11.2)	450 (10.5)	2.0	789 (10.8)	794 (10.9)	0.3
3 or +	234 (7.6)	344 (8.1)	1.6	561 (7.7)	567 (7.8)	0.4
Unknown	42 (1.4)	34 (8)	5.5	72 (10)	66 (9)	0.9
Native	1929 (63)	2468 (57.7)	10.9	4411 (60.2)	4355 (59.6)	1.4
Antepartum haemorrhage	615 (20.1)	819 (19.1)	2.4	1430 (19.5)	1404 (19.2)	0.8
Admission for preterm labour/contraction	1388 (45.3)	2053 (48)	5.3	3436 (46.9)	3411 (46.7)	0.5
PPROM	595 (19.4)	1240 (29)	22.5	1736 (23.7)	1836 (25.1)	3.3
Preeclamp/Eclamp/HELLP	536 (17.5)	617 (14.4)	8.5	1211 (16.5)	1190 (16.3)	0.7
Congenital anomalies	67 (2.2)	78 (1.8)	2.6	137 (1.9)	138 (1.9)	0.1
Infection	221 (7.2)	402 (9.4)	8.0	566 (7.7)	613 (8.4)	2.4
Diagnosis of IUGR	557 (18.2)	659 (15.4)	7.5	1242 (17)	1239 (17)	0.0
In-utero transfer	799 (26.1)	1348 (31.5)	12.0	2176 (29.7)	2144 (29.3)	0.9
Received magnesium Sulfate	211 (6.9)	239 (5.6)	5.4	496 (6.8)	461 (6.3)	1.9
Presentation:						
Vertex	1937 (63.3)	2769 (64.8)	3.1	4608 (62.9)	4678 (64)	2.2
Breech	842 (27.5)	1092 (25.5)	4.4	2008 (27.4)	1951 (26.7)	1.7
Transverse	86 (2.8)	144 (3.4)	3.3	225 (3.1)	231 (3.2)	0.5
Limb	26 (8)	30 (7)	1.5	60 (8)	53 (7)	1.1
Other	27 (9)	38 (9)	0.2	69 (9)	63 (9)	0.9
Mode of delivery:						
Vaginal delivery	1324 (43.3)	1807 (42.2)	2.0	3118 (42.6)	3162 (43.3)	1.4
Intrapartum caesarean section	714 (23.3)	1153 (27)	8.4	1896 (25.9)	1863 (25.5)	0.9
Prelabour caesarean section	1022 (33.4)	1316 (30.8)	5.6	2308 (31.5)	2285 (31.3)	0.6
Apgar score <7	636 (20.8)	608 (14.2)	17.4	1277 (17.4)	1198 (16.4)	2.8
Inborn	2537 (82.9)	3991 (93.3)	32.6	6549 (89.4)	6602 (90.3)	2.9
Delivery > 1 day of admission	933 (30.5)	672 (15.7)	35.6	1621 (22.1)	1576 (21.6)	1.4
Region						

Belgium: Flanders	291 (9.5)	421 (9.8)	1.1	673 (9.2)	688 (9.4)	0.8
Denmark: Eastern	163 (9.2)	688 (9.4)	7.6	347 (4.7)	328 (4.5)	1.2
Estonia	37 (1.2)	113 (2.6)	10.5	129 (18)	147 (2)	1.9
France: Northern	157 (5.1)	136 (3.2)	9.8	282 (3.9)	268 (3.7)	1.0
France: Burgundy	28 (0.9)	61 (1.4)	4.7	79 (1.1)	81 (1.1)	0.3
France: Ile-de-France	421 (13.8)	395 (9.2)	14.2	830 (11.3)	804 (11)	1.1
Germany: Hesse	147 (4.8)	408 (9.5)	18.4	526 (7.2)	542 (7.4)	0.9
Germany: Saarland	49 (1.6)	83 (1.9)	2.5	132 (1.8)	132 (1.8)	0.1
Italy: Lazio	307 (10)	229 (5.3)	17.7	568 (7.8)	598 (8.2)	1.6
Italy: Emilia	135 (4.4)	284 (6.6)	9.8	410 (5.6)	410 (5.6)	0.1
Italy: Marche	49 (1.6)	52 (1.2)	3.3	107 (1.5)	103 (1.4)	0.5
Netherlands: East-Central	188 (6.1)	180 (4.2)	8.7	369 (5)	363 (5)	0.3
Poland: Wielkopolska	119 (3.9)	140 (3.3)	3.4	247 (3.4)	238 (3.3)	0.7
Portugal: Northern	145 (4.8)	129 (3)	9.1	282 (3.9)	284 (3.9)	0.2
Portugal: Lisbon	288 (9.4)	135 (32)	25.8	429 (5.9)	440 (6)	0.7
United Kingdom: Northern	152 (5)	254 (5.9)	4.3	405 (5.5)	395 (5.4)	0.6
United Kingdom: East Midlands	134 (4.4)	411 (9.6)	20.7	540 (7.4)	548 (7.5)	0.5
United Kingdom : Yorkshire & Humber	173 (5.6)	518 (12.1)	22.9	658 (9)	684 (9.4)	1.3
Sweden: Stockholm	76 (2.5)	166 (3.9)	8.0	308 (4.2)	258 (3.5)	3.5

NOTE: propensity score also included 4 interactions of gestaional age with apgar, caesarean section, born on same day as maternal admission and outborn.

Table S4 - In–hospital mortality and severe morbidity by receipt of selected evidence-based practices: sensitivity analyses of All-or-None composite

	In-hospital mortality (all neonatal admissions)	Severe morbidity (survivors to discharge)	Mortality or morbidity (all neonatal admissions)
**Without appropriate place of birth**			
Not receiving EB care, N (%)	275/4730 (5.8)	354/4324 (8.2)	629/4599 (13.7)
Receiving EB care, N (%)	397/2606 (15.2)	315/2155 (14.6)	712/2552 (27.9)
Crude RR (95% CI)	0.39 [0.32 ; 0.46]	0.51 [0.44 ; 0.6]	0.47 [0.42 ; 0.53]
Adjusted RR[1] (95% CI)	0.73 [0.6 ; 0.88]	0.81 [0.7 ; 0.94]	0.81 [0.72 ; 0.9]
Propensity weighted RR[2] (95% CI)	0.73 [0.6 ; 0.89]	0.85 [0.73 ; 0.98]	0.81 [0.72 ; 0.91]
**Without surfactant/early CPAP**			
Not receiving EB care, N (%)	274/4444 (6.2)	352/4055 (8.7)	629/4599 (14.5)
Receiving EB care, N (%)	398/2892 (13.8)	317/2424 (13.1)	715/2822 (25.3)
Crude RR (95% CI)	0.47 [0.39 ; 0.57]	0.63 [0.54 ; 0.73]	0.57 [0.5 ; 0.64]
Adjusted RR[1] (95% CI)	0.74 [0.63 ; 0.88]	0.83 [0.72 ; 0.96]	0.83 [0.74 ; 0.92]
Propensity weighted RR[2] (95% CI)	0.72 [0.6 ; 0.86]	0.89 [0.77 ; 1.04]	0.84 [0.75 ; 0.94]
**Without ANC**			
Not receiving EB care, N (%)	271/4636 (5.8)	350/4247 (8.2)	621/4518 (13.8)
Receiving EB care, N (%)	401/2700 (14.8)	319/2232 (14.3)	720/2633 (27.3)
Crude RR (95% CI)	0.41 [0.34 ; 0.48]	0.54 [0.46 ; 0.64]	0.49 [0.44 ; 0.56]
Adjusted RR[1] (95% CI)	0.74 [0.62 ; 0.87]	0.84 [0.72 ; 0.97]	0.82 [0.74 ; 0.91]
Propensity weighted RR (95% CI)	0.71 [0.6 ; 0.85]	0.91 [0.77 ; 1.07]	0.83 [0.74 ; 0.94]
**Without hypothermia prevention**			
Not receiving EB care, N (%)	433/5650 (7.7)	468/5086 (9.2)	901/5519 (16.3)
Receiving EB care, N (%)	239/1686 (14.2)	201/1393 (14.4)	440/1632 (26.9)
Crude RR (95% CI)	0.54 [0.46 ; 0.65]	0.63 [0.53 ; 0.74]	0.6 [0.53 ; 0.68]
Adjusted RR[1] (95% CI)	0.79 [0.67 ; 0.92]	0.84 [0.72 ; 0.97]	0.84 [0.76 ; 0.92]
Propensity weighted RR[2] (95% CI)	0.76 [0.62 ; 0.94]	0.88 [0.72 ; 1.07]	0.84 [0.74 ; 0.96]

NOTES: 1. Adjusted for gestational age, sex, SGA, multiple pregnancy, pregnancy complications, type of delivery, Apgar score, born on same day as maternal admission without in utero transfer and region 2. adjusted on gestational age
