# Supplementary material for: Use of evidence based practices to improve survival without severe morbidity for very preterm infants: results from the EPICE population based cohort
Source: BMJ. 2016 Jul 5;354:i2976. doi: 10.1136/bmj.i2976 (PMC4933797; doi:10.1136/bmj.i2976)
Supplement: Supplementary file 2 — Appendix 2: List of hospitals participating in EPICE study [file zeij028369.ww2_default.pdf]

Acknowledgements

We would like to acknowledge the participation of the Departments of Obstetrics and Neonatology from the following hospitals in the EPICE regions:

Belgium (Flanders): ASZ Campus Geraardsbergen, Geraardsbergen; AZ Sint Maarten, Campus Zwartzustervest, Mechelen; AZ Sint Lucas, Assebroek; AZ Heilige Familie, Rumst-Reet; Sint Jozefskliniek, Izegem; AZ Sint Jozef, Malle; Sint Augustinus - MISA, Wilrijk; Onze Lieve Vrouwziekenhuis Campus Asse, Asse; AZ Diest, Diest; AZ Zeno Campus Knokke - Heist, Knokke-Heist; AZ Groeninge, Kortrijk; Sint Jozefkliniek – Campus Bornem, Bornem; Sint Vincentiusziekenhuis, Deinze; Maria Middelares, Gent; AZ Oudenaarde, Oudenaarde; AZ Glorieux, Ronse; AZ Delta Campus Menen, Menen; AZ Sint Elisabeth, Zottegem; ZOL – Campus Sint Jan, Genk; Jessa Ziekenhuis Campus Virga Jesse, Hasselt; Sint Franciskusziekenhuis, Heusden-Zolder; Maria Ziekenhuis Noord-Limburg, Overpelt; Sint Trudoziekenhuis, Sint-Truiden; AZ Damiaan, Oostende; AZ Sint Lucas, Gent; AZ Sint Blasius, Dendermonde; AZ Delta Campus Wilgenstraat, Roeselare; UZ Brussel, Brussel; ZNA Jan Palfijn, Merksem; Sint Andriesziekenhuis, Tielt; ZNA Middelheim, Antwerpen; Imeldaziekenhuis, Bonheiden; AZ Sint Maarten – Campus Duffel, Duffel; AZ KLINA, Brasschaat; AZ Jan Portaels, Vilvoorde; Universitair Ziekenhuis Antwerpen, Edegem; UZ Leuven Campus Gasthuisberg, Leuven; UZ Gent, Gent; Sint Vincentiusziekenhuis – Campus Sint Jozef, Mortsel; AZ Alma, Eeklo; AZ Turnhout, Turnhout; Heilig Hartziekenhuis, Mol; AZ Sint Jan Campus Henri Serruys, Oostende; Ziekenhuis Maas & Kempen, Bree; Sint Vincentius Ziekenhuis, Antwerpen; AZ Vesalius – Campus Sint Jacobus, Tongeren; ASZ – Campus Aalst, Aalst; Onze Lieve Vrouwziekenhuis – Campus Aalst, Aalst; AZ Delta Campus Stedelijk Ziekenhuis, Roeselare; AZ Sint Rembert, Torhout; AZ Monica – Campus Deurne, Deurne; AZ Lokeren, Lokeren; Jan Ypermanziekenhuis, Ieper; AZ Sint Elisabeth, Herentals; AZ Sint Jan, Brugge; AZ Jan Palfijn, Gent; AZ Sint Augustinus Veurne, Veurne; RZ Sint Maria, Halle; Heilig Hart Ziekenhuis, Leuven; AZ Nikolaas – Campus SM, Sint-Niklaas; Onze Lieve Vrouw van Lourdes Ziekenhuis Waregem vzw, Waregem; ACZA Ziekenhuis – Campus Sint Erasmus, Borgerhout; Heilig Hart, Lier; AZ Sint Dimpna, Geel; Heilig Hart, Tienen.

Estonia: Tallinn Children's Hospital, Unit of Newborns and Infants; Tallinna Children's Hospital, Paediatric Intensive Care Unit; Tartu University Hospital, Neonatal Unit; Tartu University Hospital, Paediatric Intensive Care Unit; East-Tallinn Central Hospital, Neonatal Unit; West-Tallinn Central Hospital, Neonatal Unit.

Denmark (Eastern Region): University Hospital of Copenhagen (Rigshospitalet); Hvidovre University Hospital; Herlev University Hospital; Hilleroed University Hospital; Roskilde University Hospital; Holbaek University Hospital; Naestved University Hospital; University Hospital of Southern Denmark

France (Burgundy): CH d'Autun, Autun; CH Auxerre, Auxerre; CH de Beaune, Beaune; CH William Morey, Chalon Sur Saone; Clinique de Cosne-Sur-Loire, Cosne-Sur-Loire; CH de Decize, Decize; CHU Le Bocage - Hôpital D'enfants, Dijon; Clinique Sainte-Marthe, Dijon; Site Hospitalier Foch, Le Creusot; CH Les Chanaux, Macon; CH de Nevers, Nevers; CH Les Charmes, Paray Le Monial; CH de Semur-En-Auxois, Semur En Auxois; CH de Sens, Sens;

France (Ile-de-France): Hôpital Prive D'Antony, Antony; CH Victor Dupouy, Argenteuil; CH Arpajon, Arpajon; Hôpital Prive D'Athis-Mons, Athis-Mons; Hôpital Européen La Roseraie, Aubervilliers; CHI Robert Ballanger, Aulnay Sous-Bois; CH Intercommunal Des Portes de L'Oise, Beaumont Sur Oise; CHU Jean Verdier, Bondy; Clinique Ambroise Paré, Bourg-La-Reine; Hôpital Privé de Marne Chantereine, Brou Sur Chantereine; Hôpital Prive de Marne La Vallée, Bry Sur Marne; Hôpital Saint-Camille, Bry Sur Marne; Clinique de Champigny-Hôpital Paul D'Egine, Champigny Sur Marne; Hôpital

Antoine Béclère, Clamart; Hôpital Beaujon, Clichy; CH Louis Mourier, Colombes; Clinique Du Parisis, Cormeilles-En-Parisis; CH René Arbeltier, Coulommiers; CHI Créteil, Créteil; Clinique Claude Bernard, Ermont; CH Sud Essonne, Etampes - Dourdan; CH Louise Michel, Evry; Clinique de L'Essonne Evry, Evry; CMO D'Evry, Evry; Polyclinique de La Forêt, Fontainebleau; CH Fontainebleau, Fontainebleau; CH de Gonesse, Gonesse; Clinique Lambert, La Garenne-Colombes; CHG de Marne La Vallée, Lagny; Hôpital Prive de Seine-Saint-Denis, Le Blanc Mesnil; CH de Versailles-André Mignot, Le Chesnay; Hôpital Prive de Parly 2 Le Chesnay, Le Chesnay; CHU Kremlin Bicêtre, Le Kremlin-Bicêtre; Maternité Des Lilas, Les Lilas; Institut Hospitalier Franco-Britannique, Levallois Perret; Clinique Conti, L'Isle-Adam; Polyclinique Vauban, Livry Gargan; Clinique de L'Yvette, Longjumeau; CH de Longjumeau, Longjumeau; CH Mantes La Jolie, Mantes La Jolie; Hôpital Prive Jacques Cartier, Massy; CH de Meaux, Meaux; CH Marc Jacquet, Melun; Polyclinique Saint Jean, Melun; Clinique de Meudon La Foret, Meudon La Foret; CH Intercommunal de Meulan-Les Mureaux, Meulan-En-Yvelines; CH de Montereau, Montereau; CHI Le Raincy-Montfermeil, Montfermeil; Groupe Hospitalier Eaubonne-Montmorency, Montmorency; CHI André Grégoire, Montreuil; Hôpital Max Fourestier, Nanterre; CH Neuilly Courbevoie, Neuilly Sur Seine; Hôpital Américain, Neuilly Sur Seine; Clinique Sainte-Isabelle, Neuilly-Sur-Seine; Hôpital Prive Armand Brillard, Nogent Sur Marne; CH D'Orsay, Orsay; Clinique de La Muette, Paris; Clinique Jeanne D'arc, Paris; Clinique Leonard de Vinci, Paris; Clinique Sainte Thérèse, Paris; Clinique Saint-Louis, Paris; GH Armand Trousseau - La Roche-Guyon, Paris; GH Diaconesses Croix St Simon, Paris; GH Pitié-Salpêtrière, Paris; GH Saint Joseph / Notre Dame de Bon-Secours, Paris; GIH Bichat/ Claude Bernard, Paris; Hôpital Cochin-Port Royal, Paris; Hôpital Lariboisière, Paris; Hôpital Les Bluets, Paris; Hôpital Necker, Paris; Hôpital Robert Debré, Paris; Hôpital Saint-Antoine, Paris; Hôpital Tenon, Paris; Institut de Puériculture Et de Périnatalogie, Paris; Institut Mutualiste Montsouris, Paris; Maternité Sainte-Félicité, Paris; CHI Poissy/Saint-Germain-En-Laye, Poissy; CH René Dubos, Pontoise; Centre Hospitalier Léon Binet, Provins; Hôpital Prive Claude Galien, Quincy-Sous-Sénart; CH de Rambouillet, Rambouillet; Clinique Les Martinets, Rueil-Malmaison; CH Des Quatre Villes, Saint Cloud; CH de Saint Denis, Saint Denis; Hôpital Esquirol St Maurice, Saint Maurice; Clinique Saint Germain, Saint-Germain-En-Laye; Hôpital Prive Nord Parisien, Sarcelles; CH Des Quatre Villes, Sèvres; Hôpital Militaire Begin, St Mande; Clinique Gaston Métivet, St Maur Des Fosses; Clinique de L'Estrées, Stains; Hôpital Foch, Suresnes; Clinique de Tournan, Tournan-En-Brie; Hôpital Prive de L'Ouest Parisien, Trappes; Clinique Du Vert-Galant, Tremblay En France; Hôpital Prive de Versailles- Franciscaines, Versailles; CH Intercommunal, Villeneuve Saint Georges; Clinique Les Noriets, Vitry Sur Seine;

France (Northern Region): CH D'Armentières, Armentières; CH D'Arras, Arras; Clinique Bon-Secours, Arras; CH de Béthune, Béthune; Clinique Anne D'Artois, Béthune; CH de Boulogne-Sur-Mer, Boulogne Sur Mer; CH de Calais, Calais; CH de Cambrai, Cambrai; Polyclinique Sainte-Marie, Cambrai; CH de Denain, Denain; Polyclinique de La Clarence, Divion; CH de Douai, Douai; Polyclinique Villette, Dunkerque; CH de Fourmies, Fourmies; GCS Flandre Maritime, Grande Synthe; CH D'hazebrouck, Hazebrouck; CH de La Région de St Omer, Helfaut; Clinique Saint-Amé, Lambres-Les-Douai; CH du Cateau, Le Cateau Cambrésis; CH de Lens, Lens; Polyclinique de Riaumont de Lievin, Lievin; GHI CL St-Vincent de Paul, Lille; Hôpital Jeanne de Flandre, Lille; Pavillon Du Bois, Lille; CH Sambre-Avesnois, Maubeuge; Polyclinique Du Val de Sambre, Maubeuge; CHAM Site Principal, Rang Du Fliers; Maternité Paul Gellé, Roubaix; CH de Seclin, Seclin; Centre Médical Chirurgical Obstétrical Côte d'Opale, St Martin / Boulogne; Clinique Maternité du Parc, St Saulve; Clinique Du Val de Lys, Tourcoing; Hôpital Guy Chatiliez, Tourcoing; CH de Valenciennes, Valenciennes; Nouvelle Clinique Villeneuve D' Ascq, Villeneuve d'Ascq.

Germany (Hesse and Saarland): Klinik für Kinder- und Jugendmedizin & Klinik für Frauenheilkunde und Geburtshilfe, Klinikum Bad Hersfeld, Bad Hersfeld; Neonatologie, Darmstaedter Kinderkliniken & Frauenklinik, Klinikum Darmstadt, Darmstadt; Klinik für Neonatologie & Frauenklinik, Buergerhospital, Frankfurt; Klinik für Kinder- und Jugendmedizin & Klinik für Gynaekologie und Geburtshilfe, Klinikum Frankfurt Hoechst, Frankfurt; Zentrum für Kinderheilkunde & Klinik fuer Frauenheilkunde und Geburtshilfe, Universitaetsklinikum Frankfurt, Frankfurt; Klinik für Kinder- und Jugendmedizin & Frauenklinik, Klinikum Fulda, Fulda; Klinik für Kinder- und Jugendmedizin & Frauenklinik, Main-Kinzig-Kliniken, Gelnhausen; Allgemeine Paediatrie und Neonatologie & Zentrum für Frauenheilkunde und Geburtshilfe, Universitaetsklinikum Giessen, Giessen; Klinik für Kinder- und Jugendmedizin & Klinik für Gynaekologie und Geburtshilfe, Klinikum Hanau, Hanau; Kinderklinik & Klinik für Frauenheilkunde und Geburtshilfe, Klinikum Kassel, Kassel; Klinik für Kinder- und Jugendmedizin & Klinik für Frauenheilkunde und Geburtshilfe, Universitaetsklinikum Marburg, Marburg; Klinik für Kinder- und Jugendmedizin & Klinik fuer Gynaekologie und Geburtshilfe, Sana Klinikum Offenbach, Offenbach; Klinik für Kinder- und Jugendmedizin & Frauenklinik, GPR Klinikum Ruesselsheim, Ruesselsheim; Abteilung Kinder und Jugendliche & Abteilung Geburtshilfe, Dr. Horst Schmidt Kliniken, Wiesbaden; Kinderklinik & Klinik fuer Frauenheilkunde, Universitaetsklinikum des Saarlandes, Homburg/Saar; Kinderklinik & Klinik fuer Frauenheilkunde, Klinikum Saarbruecken, Saarbruecken.

Italy (Emilia Romagna): Azienda Ospedaliero-universitaria di Modena, Modena; Azienda Ospedaliero-universitaria di Bologna, Bologna; Ospedale Maggiore C.A. Pizzardi, Bologna; Azienda Ospedaliera di Reggio Emilia, Reggio Emilia; Azienda Ospedaliero-universitaria di Parma, Parma; Ospedale Infermi, Rimini; Ospedale M. Bufalini, Cesena; Azienda Ospedaliero-universitaria di Ferrara, Ferrara; Ospedale Santa Maria delle Croci, Ravenna; Ospedale Morgagni – Pierantoni, Forlì; Ospedale Guglielmo da Saliceto, Piacenza; Ospedale Civile Nuovo Santa Maria della Scaletta, Imola; Ospedale Degli Infermi, Faenza; Ospedale Santa Maria Bianca, Mirandola; Ospedale Civile Guastalla, Guastalla; Ospedale SS. Annunziata, Cento; Ospedale Umberto I, Lugo; Ospedale Unico della Val D'Arda, Fiorenzuola D'Arda; Ospedale San Secondo, Fidenza; Ospedale B. Ramazzini, Carpi; Ospedale Pavullo nel Frignano, Pavullo nel Frignano; Nuovo Ospedale Civile di Sassuolo, Sassuolo; Ospedale di Bentivoglio, Bentivoglio.

Italy (Lazio Region): Policlinico Umberto I; Policlinico A. Gemelli; Azienda Ospedaliera San Camillo; Azienda Ospedaliera San Giovanni; Azienda Ospedaliera San Filippo Neri; Ospedale Pediatrico Bambino Gesù; Ospedale Belcolle-Viterbo; Ospedale Sant'Eugenio; Ospedale SG Calibita Fatebenefratelli; Ospedale S. Pietro Fatebenefratelli; Policlinico Casilino.

Italy (Marche): Azienda Ospedaliero-universitaria Ospedali Riuniti Umberto I – G.M. Lancisi – G. Salesi, Ancona; Ospedale Generale Provinciale C.G. Mazzoni, Ascoli Piceno; Ospedale Generale Provinciale Macerata, Macerata; Azienda Ospedaliera San Salvatore, Pesaro; Ospedale A. Murri, Fermo; Ospedali Riuniti di Jesi, Jesi; Ospedale Civile E. Profili, Fabriano; Ospedale Santa Croce, Fano.

The Netherlands (Eastern & Central):

Radboudumc Nijmegen; Wilhelmina Childrens Hospital Utrecht; Canisius Wilhelmina Hospital Nijmegen; Maasziekenhuis Pantein Boxmeer; Bernhoven Hospital Uden; Gelderse Vallei Hospital Ede; Rijnstate Hospital Zevenaar; Rijnstate Hospital Arnhem; Slingeland Hospital Doetinchem; Regional Hospital Koningin Beatrix Winterswijk; Gelre Hospital Zutphen; Meander Medical Center Amersfoort; Gelre Hospital Apeldoorn; Diakonessenhuis Utrecht; Zuwe Hofpoort Hospital Woerden; Tergooi Hilversum; Hospital Rivierenland Tiel; St Antonius Hospital Nieuwegein; TweeSteden Hospital Tilburg; St Elisabeth Hospital Tilburg; Deventer Hospital Deventer; Tergooi Hospital Blaricum.

Poland: Department of Neonatology, Poznan University of Medical Sciences (III level); Szpital Wojewódzki w Poznaniu (II level); Szpital Miejski im. Franciszka Raszei w Poznaniu (II level); Specjalistyczny Zespół Opieki Zdrowotnej nad Matką i Dzieckiem w Poznaniu, Szpital Św. Rodziny (II level); Wojewódzki Szpital Zespolony im. L. Perzyny w Kaliszu (II level); Wojewódzki Szpital Zespolony w Koninie (II level); Wojewódzki Szpital Zespolony w Lesznie (II level); Szpital Specjalistyczny im. Stanisława Staszica w Pile (II level); Zespół Zakładów Opieki Zdrowotnej w Ostrowie Wielkopolskim (III level); Szpital Powiatowy im. Prof. Romana Drewsa w Chodzieży (I level); Zespół Zakładów Opieki Zdrowotnej w Czarnkowie (I level); Samodzielny Publiczny Zespół Opieki Zdrowotnej w Gostyniu (I level); Zespół Opieki Zdrowotnej w Gnieźnie (I level); Samodzielny Publiczny Zakład Opieki Zdrowotnej w Grodzisku Wielkopolskim (I level); Zespół Zakładów Opieki Zdrowotnej w Jarocinie (I level); Samodzielny Publiczny Zakład Opieki Zdrowotnej w Kępnie (I level); Samodzielny Publiczny Zakład Opieki Zdrowotnej w Kole (I level); Samodzielny Publiczny Zakład Opieki Zdrowotnej w Kościanie (I level); Samodzielny Publiczny Zakład Opieki Zdrowotnej w Krotoszynie (I level); Samodzielny Publiczny Zakład Opieki Zdrowotnej im. K. Hołygi w Nowym Tomyślu (I level); Samodzielny Publiczny Zakład Opieki Zdrowotnej w Obornikach (I level); Zespół Zakładów Opieki Zdrowotnej w Ostrzeszowie (I level); Pleszewskie Centrum Medyczne w Pleszewie (II level); Szpital Powiatowy w Rawiczu (I level); Samodzielny Publiczny Zakład Opieki Zdrowotnej w Międzychodzie (I level); Samodzielny Publiczny Zakład Opieki Zdrowotnej w Słupcy (I level); Szpital w Śremie (I level); Samodzielny Publiczny Zakład Opieki Zdrowotnej im. Dr J. Dietla w Środzie Wielkopolskiej (I level); Samodzielny Publiczny Zakład Opieki Zdrowotnej w Szamotułach (I level); Szpital Powiatowy im. Jana Pawła II w Trzciance (I level); Samodzielny Publiczny Zakład Opieki Zdrowotnej w Turku (I level); Zespół Opieki Zdrowotnej w Wągrowcu (I level); Samodzielny Publiczny Zakład Opieki Zdrowotnej w Wolsztynie (I level); Szpital Powiatowy we Wrześni (I level); Szpital Powiatowy w Wyrzysku (I level); Szpital Powiatowy im. A. Sokołowskiego w Złotowie (I level).
Portugal (Northern Region); Centro Hospitalar de Entre o Douro e Vouga, E.P.E. - Hospital de São Sebastião; Centro Hospitalar de Trás-os-Montes e Alto Douro, E.P.E. - Hospital São Pedro; Centro Hospitalar de Vila Nova de Gaia/Espinho, E.P.E. - Unidade II; Centro Hospitalar do Alto Ave, E.P.E. - Unidade de Guimarães; Centro Hospitalar do Médio Ave, E.P.E. - Unidade de Famalicão; Centro Hospitalar do Porto, E.P.E. - Maternidade Júlio Dinis; Centro Hospitalar do Tâmega e Sousa, E.P.E. - Hospital Padre Américo; Centro Hospitalar Póvoa de Varzim - Vila do Conde, E.P.E. - Unidade da Póvoa de Varzim; Centro Hospitalar São João, E.P.E. - Hospital São João; Hospital de Braga; Unidade Local de Saúde de Matosinhos, E.P.E. - Hospital Pedro Hispano; Unidade Local de Saúde do Alto Minho, E.P.E. - Hospital de Santa Luzia; Unidade Local de Saúde do Nordeste, E.P.E. - Unidade de Bragança.
Portugal (Lisbon and Tagus Valley Region) : Centro Hospitalar Barreiro Montijo, E.P.E.- Hospital Nossa Senhora do Rosário; Centro Hospitalar de Lisboa Central, E.P.E. - Hospital Dona Estefânia; Centro Hospitalar de Lisboa Central, E.P.E. - Maternidade Alfredo da Costa; Centro Hospitalar de Lisboa Ocidental, E.P.E. - Hospital de São Francisco Xavier; Centro Hospitalar de Setúbal, E.P.E. - Hospital São Bernardo; Centro Hospitalar do Médio Tejo, E.P.E. - Hospital Doutor Manoel Constâncio; Centro Hospitalar do Oeste - Unidade de Caldas da Rainha; Centro Hospitalar do Oeste - Unidade de Torres Vedras; Centro Hospitalar Lisboa Norte, E.P.E. - Hospital Santa Maria; Hospital Cuf Descobertas; Hospital da Luz; Hospital de Cascais Dr. José de Almeida; Hospital de Santarém, E.P.E.; Hospital Garcia de Orta, E.P.E.; Hospital Lusíadas Lisboa; Hospital Professor Doutor Fernando Fonseca, E.P.E.; Hospital Vila Franca de Xira.

Sweden (Stockholm): Department of Obstetrics and Gynecology, Danderyd Hospital, Stockholm; BB Stockholm AB, Danderyd Hospital, Stockholm; Department of Obstetrics and Gynecology, Karolinska University Hospital (units in Solna and Huddinge), Stockholm; Department of Obstetrics and Gynecology, Sodersjukhuset (Stockholm South General Hospital), Stockholm; Department of Obstetrics and Gynecology, Sodertalje Hospital; Department of Neonatal Medicine, Karolinska University Hospital (units in Danderyd, Solna and Huddinge), Stockholm; Sachs' Children and Youth Hospital, Stockholm.
United Kingdom (Northern Region): Royal Victoria Infirmary Newcastle upon Tyne; James Cook University Hospital Middlesbrough; North Tees University Hospital Stockton; Sunderland Royal Hospital; Wansbeck Hospital Ashington; Queen Elizabeth Hospital Gateshead; North Tyneside General Hospital; South Tyneside General Hospital; Cumberland Infirmary Carlisle; West Cumberland Infirmary.
United Kingdom (East Midlands - Yorkshire & Humber): Chesterfield Royal Hospital;Bassetlaw District General Hospital;Kings Mill Hospital;Royal Derby Hospital;Nottingham City Hospital;Nottingham Queen's Medical Centre;Lincoln County Hospital;Boston Pilgrim Hospital;University Hospitals of Leicester (LGH + LRI);Kettering General Hospital;Northampton General Hospital;Grimsby Diana Princess of Wales Hospital;Scunthorpe General Hospital;Barnsley District General Hospital;Rotherham District General Hospital;Doncaster Royal Infirmary;Jessop Wing Sheffield;Airedale District General Hospital;Bradford Royal Infirmary;Dewsbury District General Hospital;Halifax Calderdale Royal Infirmary;Harrogate District General Hospital;Hull Royal Infirmary;Leeds General Infirmary; Leeds St James's;Scarborough District General Hospital;York District Hospital;Wakefield Pinderfields General Hospital.
